# Broad and robust T cell immunity compensates the population immune barrier against antibody-escaping Omicron variants

**DOI:** 10.3389/fimmu.2026.1731340

**Published:** 2026-01-30

**Authors:** Yuanyuan Guo, Peipei Guo, Jinmin Tian, Yeerzati Tuluhongtayi, Rui Song, George F. Gao, Xin Zhao, Jun Liu

**Affiliations:** 1 Department of Epidemiology, School of Public Health, Cheeloo College of Medicine, Shandong University, Jinan, China; 2 Strategic Pandemic Preparedness Action and Research Center (SPARC), Guangzhou National Laboratory, Guangzhou, China; 3 Beijing Ditan Hospital, Capital Medical University, Beijing, China; 4 Laboratory of Pathogen Microbiology and Immunology, Institute of Microbiology, Chinese Academy of Sciences (CAS), Beijing, China

**Keywords:** immune evasion, neutralizing antibodies, Omicron variants, population immune barrier, SARS-CoV-2, T cell immunity

## Abstract

**Background:**

Continuous SARS-CoV-2 Omicron emergence poses challenges to immune protection from the previous infection/vaccination in the population. While neutralizing antibodies serve as a key immune protection indicator, their cross-protective effect against novel variants remains limited. However, T cell immunity may confer more durable and broad-spectrum protection.

**Methods:**

We evaluated immune dynamics in four Chinese cohorts comprising BF.7/BA.5.2, XBB, and JN.1 convalescents, plus tetravalent recombinant protein vaccine recipients. Neutralizing antibodies were assessed against nine variants spanning the emerging evolutionary spectrum. T cell responses were characterized using variant-specific peptide pools. Antigenic relationships were analyzed through multidimensional scaling-based cartography.

**Results:**

BF.7/BA.5.2 convalescents exhibited progressive antibody evasion, with fold-changes against heterologous variants increasing from 4–12-fold initially to > 20-fold at 6 months. XBB convalescents maintained stable short-term responses, while JN.1 convalescents showed superior cross-reactivity against descendant lineages. BA.3.2 demonstrated maximal immune evasion across all groups, occupying the most distant antigenic position. In contrast, T cell immunity exhibited remarkable stability and cross-reactivity, maintaining elevated levels at 6 months with balanced responses against all tested variants. The tetravalent vaccine induced broad-spectrum T cell responses comparable to natural infection, and elicited cross-neutralizing antibody responses against different Omicron variants.

**Discussion:**

Our study reveals SARS-CoV-2 variant-specific antibody escape compensated by stable cross-reactive T cell responses. In the context of continued viral evolution, stimulating robust T cell immune responses may be critical to achieve a high population immune barrier against future coronaviruses/variants. These findings emphasize the necessity of comprehensive immune evaluation integrating both humoral and cellular components and provide scientific foundations for optimizing vaccine strategies and immune surveillance systems to address emerging viruses and their variants.

## 1 Introduction

Since the outbreak in late 2019, severe acute respiratory syndrome coronavirus 2 (SARS-CoV-2) has undergone continuous mutation and evolution, generating diverse variants with enhanced transmissibility and immune evasion capabilities (1). Viral evolution has progressed from early variants of concern (e.g., Delta) to the globally dominant Omicron variants, including BA.1, BA.2, BA.4/5, BF.7, XBB and BA.2.86/JN.1, creating a complex evolutionary landscape that poses ongoing challenges to global public health (2–5). Currently, descendants of JN.1 remain dominant globally, including the KP.3 series, the recombinant variant XEC, and rapidly spreading emergent variants such as NB.1.8.1, the LP.8.1 series, and XFG (6–9). Notably, BA.3.2, a highly divergent variant carrying over 50 mutations relative to BA.3, has emerged with unique spike (S) protein conformational changes that substantially enhance immune evasion despite limited global circulation (10). The continued emergence of such antigenically-distinct variants underscores the dynamic nature of SARS-CoV-2 evolution and the need for comprehensive immune surveillance.

Understanding immune responses to these evolving variants is critical for pandemic management (11). Serum neutralizing antibodies (NAbs) constitute important immune defenses against SARS-CoV-2 infection, yet their levels decline rapidly months following infection or vaccination. SARS-CoV-2 NAbs exhibit significant decay 6–8 months post-recovery. Although severe patients initially generate higher antibody titers, decay kinetics remain comparable to those observed in mild cases (12, 13). Vaccination-induced NAbs demonstrate similar time-dependent attenuation, with substantial decreases observed within 3–6 months across various vaccine platforms, including inactivated, mRNA, and adenoviral vectors (14–17). Critically, emerging variants demonstrate increasing NAb evasion capabilities. Studies indicate that NAb titers against BQ.1.1 and XBB.1 variants are 3–8 fold and 8–21 fold lower than those against BA.5, respectively (18, 19). BA.2.86, and JN.1 lineage variants exhibit 2–5 fold reduced NAb titers compared with early Omicron variants such as XBB.1.5 and display broad resistance to most therapeutic monoclonal antibodies (20–22). The widespread humoral immune evasion by emerging variants substantially diminishes NAb-based protection, culminating in breakthrough infections and reinfections even among highly vaccinated populations.

Conversely, T cell immunity confers more durable and broad-spectrum protection than humoral responses against SARS-CoV-2 (23, 24). Studies consistently demonstrate that virus-specific T cell cross-reactivity against variants is well-preserved (25, 26). Among COVID-19 convalescents, 70–90% of CD4^+^ and CD8^+^ T cells retain reactivity against various Omicron variants, including the highly mutated BA.2.86 and JN.1 variants (27–29). This cross-reactivity stems from the diversity and conservation of T cell epitopes, which recognize diverse viral proteins including S, nucleocapsid (N), and membrane (M) proteins, thereby limiting the impact of individual mutations (30–32). Longitudinal studies of SARS convalescents revealed that SARS-CoV-specific T cells remained detectable years post-infection and exhibited strong cross-reactivity against SARS-CoV-2 (33–36). Additionally, although NAbs in SARS-CoV-2 convalescents declined substantially 12 months post-recovery, specific T cell responses persisted robustly and demonstrated cross-reactivity against Alpha, Beta, Gamma, and Delta variants (37, 38). This prolonged T cell memory provides crucial protection against novel variants. Evidence indicates that T cell cross-reactivity provides critical protection despite ineffective NAbs. Epidemiological data demonstrate that although infection rates have increased due to continuously emerging variants, protection against severe disease and death from reinfection remains high, largely attributable to sustained T cell immunity (39, 40). These findings underscore the importance of incorporating T cell responses into assessments of population immunity and vaccine effectiveness.

Since late 2022, the epidemic strains of SARS-CoV-2 involved a distinct succession pattern of Omicron lineage variants, from BF.7/BA.5.2 to XBB and then to JN.1 (41). This unique epidemiological trajectory provides an exceptional opportunity to examine immune characteristics across different exposure histories. However, systematic evaluation of humoral and cellular immune response dynamics, breadth, and persistence in this context remains limited. Given ongoing viral evolution and continued variant emergence, a comprehensive investigation of variant-specific immune impacts holds substantial scientific and clinical importance (42). We hypothesized that despite variant-specific antibody decay, T cell immunity maintains broader cross-reactivity, establishing a compensatory population immune barrier. This study aims to elucidate humoral-cellular interplay across SARS-CoV-2 infections and vaccination.

Conclusively, our systematic evaluation of serum neutralization activity and T cell responses in BF.7/BA.5.2, XBB, and JN.1 convalescent cohorts, as well as recombinant protein vaccine recipients, elucidate cross-reactive response patterns and temporal dynamics of immune profile following variant-specific infections and their relationship to viral antigenic evolution. We found that while neutralizing antibody responses exhibit variant-specific attenuation patterns and gradually evade immune responses against variants with more distant antigenicity, T cell immunity maintains broader cross-reactivity within the Omicron lineage, thus consolidating the population immune barrier against the virus variants with antibody-escaping features. These findings provide essential data for SARS-CoV-2 immune surveillance, variant risk assessment, and vaccine strategy optimization, and also contribute novel insights into viral immune evasion mechanisms and host-pathogen immune equilibrium.

## 2 Materials and methods

### 2.1 Study design and participants

Four distinct participant cohorts were recruited between December 2022 and August 2024 (**Supplementary Table 1**). The BF.7/BA.5.2 convalescent cohort comprised adults who recovered from SARS-CoV-2 infection in Beijing between December 2022 and January 2023. Blood samples were collected 14–28 days post-recovery (n=45) and 6 months post-recovery (n=31). The XBB convalescent cohort included individuals infected with XBB lineage variants who recovered between June and July 2023. Blood samples were collected at 7 days (n=25) and 14–28 days (n=25) post-recovery. The JN.1 convalescent cohort consisted of individuals infected with the JN.1 variant between April and August 2024, with blood samples collected 14–28 days post-recovery (n=18). The vaccination cohort comprised people of the recombinant protein vaccine (a tetravalent COVID-19 vaccine containing trimeric spike proteins from SARS-CoV-2 Alpha, Beta, Delta, and Omicron BA.1 variants (43)), with blood samples collected at baseline (Day 0) and 21 days post-vaccination (n=15). This study employed convenience sampling during consecutive peak periods of the epidemic, and the sample size was consistent with established practices for exploratory immunological research on COVID-19.

All SARS-CoV-2 infections were confirmed by quantitative reverse transcription-polymerase chain reaction (qRT-PCR) or antigen testing of throat swabs. Infecting strains were determined based on local epidemiological surveillance data during transmission peaks when specific variants achieved clear dominance (> 90% prevalence; **Supplementary Figure 1**). All participants reported no respiratory symptoms within 3 months prior to their most recent SARS-CoV-2 infection. The study protocol received approval from the ethics committees of the National Institute for Viral Disease Control and Prevention, Chinese Center for Disease Control and Prevention. All participants provided written informed consent.

### 2.2 Sample collection and processing

Peripheral venous blood samples were collected at predetermined time points. For serum preparation, blood samples were allowed to clot at room temperature for 30 min, then centrifuged at 3,000 rpm for 15 min. Serum was then aliquoted and stored at −80 °C until test. For peripheral blood mononuclear cell (PBMC) isolation, blood samples were collected in heparinized tubes and processed within 4 h after blood collection. PBMCs were isolated using Ficoll density gradient centrifugation (37). Blood samples were diluted and carefully layered onto Ficoll-Paque lymphocyte separation medium, then centrifuged at 800 g for 30 min at room temperature with acceleration/deceleration setting of 3/1. The PBMC layer at the plasma-medium interface was harvested, washed 1–2 times with RPMI 1640 medium (1,500 rpm, 10 min), resuspended, and counted. Fresh PBMCs were used immediately for T cell assays or cryopreserved in liquid nitrogen using fetal bovine serum (FBS) containing 10% dimethyl sulfoxide (DMSO).

### 2.3 Expression plasmid construction

Spike protein expression plasmids were constructed for key variants, including prototype (ancestral strain, refer to Wuhan-Hu-1), BF.7, XBB.1.5, JN.1, KP.3, XEC, NB.1.8.1, LP.8.1.1 and BA.3.2. Codon-optimized spike protein ectodomain sequences were designed based on variant-specific sequences, with T4 fibritin trimerization domains fused to the C-terminus to enhance protein stability, as previously described (44, 45). Site-directed mutagenesis was performed to introduce characteristic mutations for each variant, and all constructs were verified by DNA sequencing. Spike protein expression vectors utilized mammalian cell expression systems containing CMV promoters, multiple cloning sites, and SV40 poly-A signals. Appropriate tag sequences were incorporated at N- or C-termini to facilitate downstream purification and detection.

### 2.4 VSV pseudovirus production

Pseudoviruses were generated using the vesicular stomatitis virus (VSV) pseudo typing system (46). HEK-293T cells were seeded in 10 cm culture dishes to reach 70–80% confluence, then transfected with the corresponding spike protein expression plasmid using lipofectamine transfection reagent. Twenty-four hours post-transfection, cells were infected with G protein-deficient VSV (VSV-ΔG-GFP) at a multiplicity of infection (MOI) of 3–5. Two hours post-infection, cells were extensively washed with phosphate-buffered saline (PBS) to remove residual inoculum. The culture medium was then replaced with Dulbecco’s Modified Eagle Medium (DMEM) containing 10% FBS, and anti-VSV-G antibody was added to a final concentration of 10 μg/mL. After 24 h, supernatants were harvested, filtered through 0.45 μm membranes to remove cellular debris, and stored in aliquots at −80 °C. Pseudovirus titers were determined using Vero E6-ACE2 cells. Infectious units were quantified by enumerating infected cells under fluorescence microscopy, with titers expressed as transducing units (TU) per mL.

### 2.5 Pseudovirus neutralization assay

Pseudovirus neutralization assays were performed following established protocols. Serum samples were heat-inactivated at 56 °C for 30 min to inactivate complement, then serially diluted 2-fold starting from 1:20. Equal volumes of diluted sera were mixed with standardized pseudovirus (approximately 1,000 TU) in 96-well plates and incubated at 37 °C with 5% CO_2_ for 1 h to allow antibody-virus binding. Subsequently, 100 μL of serum-virus mixtures were transferred to 96-well plates pre-seeded with Vero E6-ACE2 cells. Following approximately 15 h of culture, TU were quantified using a CQ1 confocal imaging cytometer. The 50% pseudovirus neutralization titer (pVNT_50_) was calculated by fitting the dose-response curve using four-parameter logistic regression analysis. Samples with pVNT_50_ values below the detection threshold (< 20) were classified as seronegative and assigned a value of 10 for geometric mean titer (GMT) calculation. Immune escape fold change was calculated as the ratio of reference variant pVNT_50_ (typically the homologous infection strain) to target variant pVNT_50_.

### 2.6 Peptide pool synthesis and *in vitro* PBMC culture

Overlapping peptide pools covering S1 (101 peptides) and RBD (30 peptides) regions were synthesized based on variant-specific spike protein sequences. Peptides were 15–18 amino acids in length with 10 amino acid overlaps, adjusted according to the mutation patterns in different Omicron variants relative to the prototype sequence. Peptide purity exceeded 95%. Peptides were dissolved in DMSO to prepare 20 mg/mL stock solutions and diluted to working concentrations in culture medium before use. Convalescent PBMCs were cultured for 9 days using S1 peptide pools corresponding to their infecting variants, i.e., BF.7/BA.5.2 convalescents with BF.7/BA.5.2-S1 peptide pools, XBB convalescents with XBB-S1 peptide pools, and vaccine recipients with BA.5.2-S1 peptide pools (reflecting the vaccine’s early Omicron components). Subsequently, cultured cells were used to test specific T cells against S1 and RBD peptide pools from representative variants, including homologous strains.

### 2.7 ELISpot assay

Antigen-specific T cell responses were assessed using IFN-γ ELISpot kits (BD Biosciences, Franklin Lakes, NJ, USA). Pretreated 96-well ELISpot plates were coated with anti-human IFN-γ monoclonal antibody overnight at 4 °C. Following PBS washed, cells were blocked with RPMI 1640 complete medium containing 10% FBS. Cultured PBMCs (10^5^ cells/well) were seeded into pre-coated plates with corresponding peptide pools (final concentration 2 μg/mL). Negative control (medium only), positive control (phorbol 12-myristate 13-acetate, PMA, 5 μg/mL), and variant peptide pool stimulation groups were established. After incubation at 37 °C with 5% CO_2_ for 18–24 h, cells were discarded and plates washed with water-Tween solution. Biotinylated detection antibodies were added and incubated at room temperature for 2 h. After PBS-Tween washes, streptavidin was added and incubated at room temperature for 1 h. Color development was performed using a substrate solution and terminated with deionized water. Following plate drying, spots were enumerated using an ELISpot reader (CTL Corp, Ohio, USA). Results were expressed as spot-forming cells per million PBMCs (SFCs/10^6^ PBMCs).

### 2.8 Intracellular cytokine staining (ICS)

PBMCs were adjusted to 1×10^6^ cells/mL and stimulated with corresponding peptide pools (2 μg/mL) at 37 °C with 5% CO_2_ for 1 h. Protein transport inhibitors brefeldin A (10 μg/mL) and monensin (2 μM) were then added, with incubation continued for 9–12 h. Post-stimulation, cells were harvested and stained with Live/Dead APC-Cy7 dye for viability assessment. Surface staining was performed using anti-CD3-FITC, anti-CD4-PerCP-Cy5.5, and anti-CD8-BV510 antibodies with incubation at 4 °C for 30 min. Cells were then fixed and permeabilized, followed by intracellular staining with anti-IFN-γ-PE-Cy7, anti-TNF-α-PE, and anti-IL-2-APC at 4 °C for 30 min. Following washes, cells were analyzed using a flow cytometer (FACSAria II, BD Biosciences), with at least 10,000 lymphocyte events collected per sample. Data analysis was performed using FlowJo software. Results were expressed as percentages of cytokine-positive cells within corresponding T cell subsets following the gating strategy as described previously (**Supplementary Figure 2**).

### 2.9 Antigenic cartography

Antigenic cartography was constructed based on neutralization antibody data. Multidimensional scaling (MDS) was employed to reduce the high-dimensional neutralization data to a two-dimensional space, where antigenic distances correspond to immunological distances. Antigenic distance was defined as log_2_ (relative neutralization titer), with relative neutralization titer calculated as the ratio of test strain pVNT_50_ to reference strain pVNT_50_. pVNT_50_ values were first converted to antigenic distances, then MDS was used to project viral variants and serum samples into two-dimensional antigenic maps such that Euclidean distances between points optimally reflected actual antigenic relationships. Analysis was performed using the Racmacs package (https://acorg.github.io/Racmacs/) in R. Serum clustering in maps represents samples with similar antibody recognition profiles, while viral strain positions reflect their antigenic similarities. Radar plot analysis visualized overall neutralization patterns of different sera against variant panels. Neutralization profiles were plotted using GMT values for each variant as coordinate axis. Overall neutralization strength was quantified by area calculation, with cross-reactive response pattern assessed through shape analysis.

### 2.10 Sequence alignment and phylogenetic analysis

All variant sequences used for phylogenetic and antigenic analyses were obtained from NCBI Virus database (https://www.ncbi.nlm.nih.gov/labs/virus/) (**Supplementary Table 2**). Multiple sequence alignment and phylogenetic analysis were performed using the Clustal W algorithm. Phylogenetic trees were constructed based on full-length spike protein amino acid sequence using MEGA X.

### 2.11 Quantification and statistical analysis

All analyses were conducted using R software (version 4.3.0) and GraphPad Prism software (version 9.5). Neutralizing antibody titers were expressed as GMT with 95% confidence intervals (CI), and T cell responses were expressed as medians with interquartile ranges (IQR). For cross-sectional comparisons among multiple groups or variants, Friedman tests (non-parametric test for repeated measures or matched data) were used, followed by Dunn’s multiple comparison test for pairwise comparisons. For longitudinal paired data (e.g., 14–28 days vs. 6 months in the BF.7/BA.5.2 cohort), the Wilcoxon matched-pairs signed-rank test was employed. Escape fold changes were calculated as ratios of reference strain GMT to test strain GMT. Significance levels were denoted as “ns” (non-significant); *P < 0.05; **P < 0.01; ***P < 0.001; ****P < 0.0001. Specific statistical tests used for each analysis are indicated in the corresponding figure legends.

## 3 Results

### 3.1 Phylogenetic analysis reveals evolutionary dynamics of antigenicity of emerging Omicron variants

**Figure 1:**
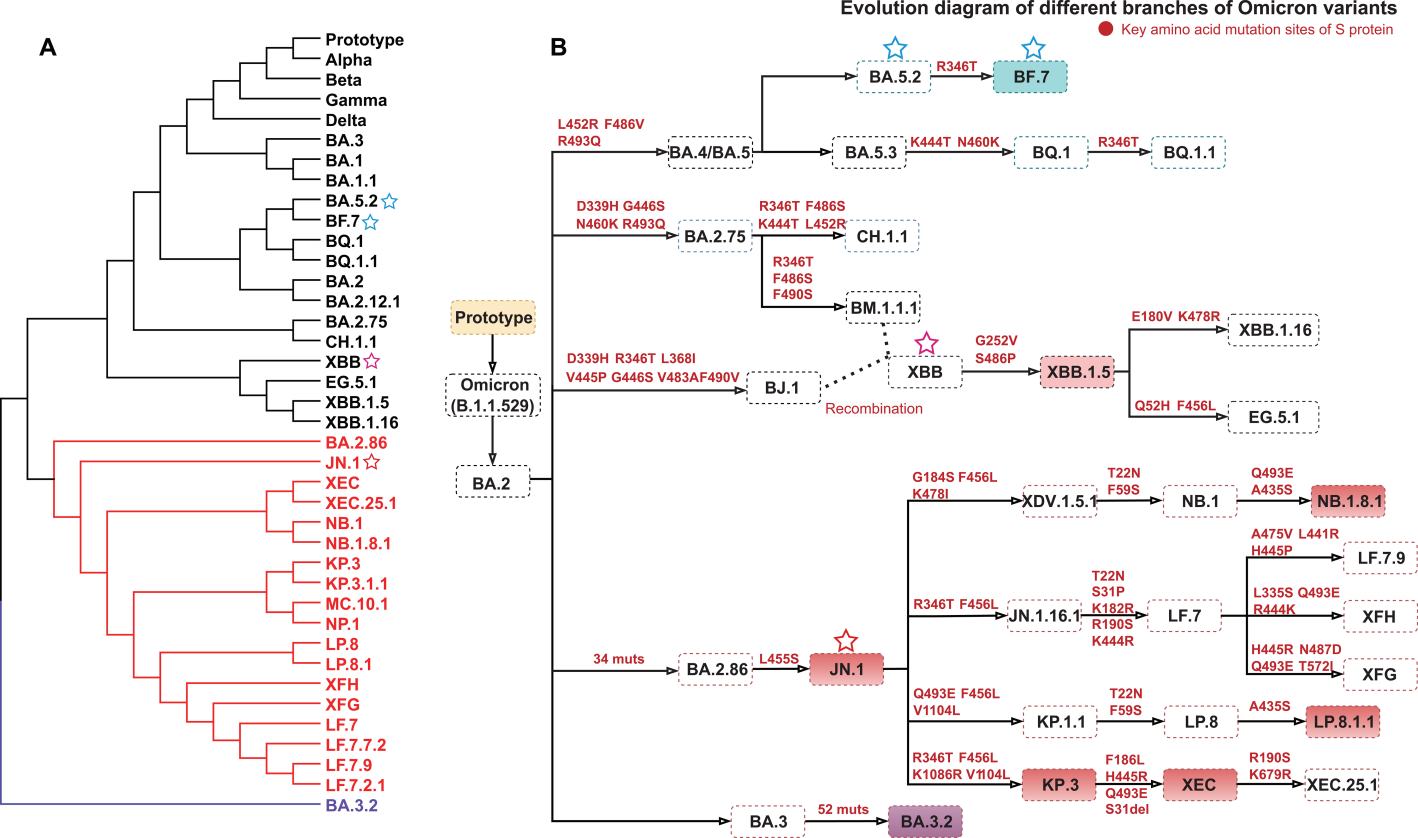
Phylogenetic analysis and mutation characterization of SARS-CoV-2 Omicron variants. **(A)** Phylogenetic tree of major SARS-CoV-2 variants. The phylogenetic tree was constructed using a maximum likelihood approach based on full-length spike protein amino acid sequences. Branch lengths correspond to evolutionary distances (scale bar indicates substitutions per site). Red branches represent the BA.2.86/JN.1 lineage and its descendants, while purple branches represent the independent evolutionary branch of BA.3.2. **(B)** Detailed evolutionary trajectory of Omicron variants. The diagram illustrates the evolutionary path from the ancestral strain through Omicron (B.1.1.529) to major variants and representative derivatives. Arrows indicate evolutionary progression and chronological order. Mutations marked in red represent known key immune escape sites or receptor binding sites. Numbers indicate cumulative mutation counts (Muts). Compared with BA.2, the BA.2.86 variant harbors 34 spike protein mutations, and BA.3.2 carries 52 mutations relative to BA.3. Variant nomenclature follows GISAID and Pangolin classification systems. The pentagram indicates the variant of the convalescent infection used in this study. The solid-colored boxes in panel B indicate the strains for which neutralizing antibodies were detected in this study.

To characterize the evolutionary relationships among emerging SARS-CoV-2 Omicron variants, we conducted maximum likelihood phylogenetic reconstruction based on full-length spike protein sequences, which revealed the complex evolutionary trajectory from prototype to contemporary Omicron variants (**Figure 1A**). The evolutionary trajectory diagram demonstrated a detailed evolutionary trajectory step-wise progression of Omicron variants (**Figure 1B**). BF.7/BA.5.2, as descendants of BA.4, dominated initial Omicron epidemic wave in China during late 2022. This was followed by the successive emergence and spread of recombinant XBB lineages and the JN.1 series. Driven by the progressive accumulation of mutations in the spike protein, JN.1 and its descendant variants (e.g., NB.1.8.1, LP.8.1.1, and XEC) now achieve global ascendancy, a trend that underscores their rapid emergence and expansion under evolutionary selective pressure. The evolutionary diagram revealed that BA.2.86 harbored 34 spike protein mutations compared with BA.2, while BA.3.2 carried 52 mutations relative to BA.3, underscoring the substantial genetic divergence of these variants. Mutational analysis revealed striking patterns of convergent evolution, whereby phylogenetically distinct lineages independently acquired identical key mutations. Multiple variants convergently evolved the R346T mutation, appearing across BF.7, XBB, and KP.3 variants. Similarly, mutations at positions L452 (L452R or L452Q) and F486 (F486P or F486V) were repeatedly selected in later-emerging variants, including BF.7, BQ.1, and JN.1, indicating strong positive selection for these amino acid changes. The systematic accumulation of mutations, particularly in antigenic regions of the spike protein, established the basis for evaluating their impact on neutralizing antibody responses and T cell cross-reactivity in different convalescent populations.

### 3.2 BF.7/BA.5.2 convalescents exhibited reduced cross-neutralizing responses against escape variants with sustained T cell immunity

**Figure 2:**
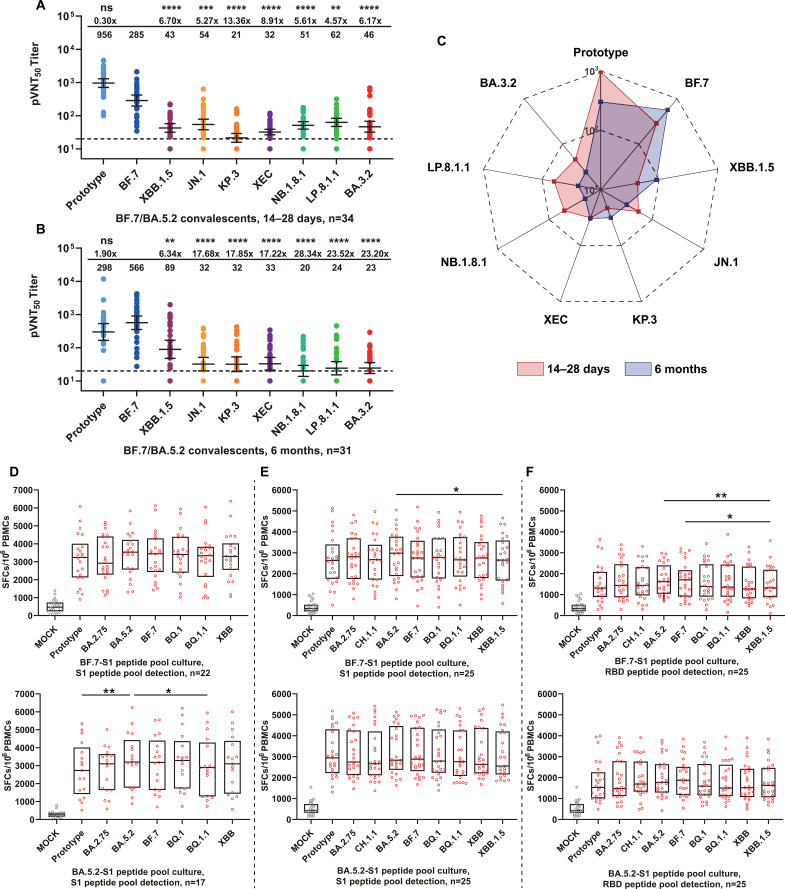
Neutralizing antibody kinetics and T cell cross-reactivity in BF.7/BA.5.2 convalescents. **(A, B)** Serum neutralizing antibody titers determined by pseudovirus assays. **(A)** The 50% pseudovirus neutralization titer (pVNT_50_) results for sera collected 14–28 days post-recovery (n=34) and **(B)** 6 months post-recovery (n=31) against multiple variants. Individual samples are represented as dots, with lines indicating geometric mean titers (GMT) and 95% confidence intervals (CI). The horizontal dashed line marks the detection threshold (1:20). Values above panels indicate immune escape fold changes relative to the homologous BF.7 strain and GMT for each group. Individual-level temporal trajectories for longitudinal cohorts are shown in **Supplementary Figure 3**. **(C)** Neutralizing antibody response profiles by radar plot analysis. Comparative neutralization patterns against different variants in BF.7/BA.5.2 convalescents 14–28 days (red shading) and 6 months (blue shading) post-recovery. Radial axes represent logarithmic GMT scales. **(D–F)** T cell responses assessed by IFN-γ ELISpot. Variant-specific T cell reactivity of PBMCs against S1 peptide pools at 14–28 days post-recovery **(D)**, at 6 months for S1 peptides **(E)**, and for RBD peptides at 6 months **(F)**. PBMCs were cultured for 9 days with BF.7 and BA.5.2 S1 peptide pools, respectively, with detection of IFN-γ-secreting cells following S1 **(D, E)** or RBD **(F)** peptide stimulation. Data points represent individual samples, with box plots displaying median, interquartile range. MOCK represents unstimulated negative controls. Results are expressed as spot-forming cells per million PBMCs (SFCs/10^6^ PBMCs). BF.7/BA.5.2 convalescents demonstrated time-dependent neutralizing antibody decline, with markedly enhanced immune evasion by heterologous variants after 6 months. Conversely, T cell responses remained robust after 6 months and exhibited cross-reactivity, confirming the persistence and breadth of cellular immunity. Statistical analyses employed Friedman tests, with pairwise comparisons using Dunn’s multiple comparison procedures unless otherwise noted. The “ns” denotes non-significant differences; *P < 0.05; **P < 0.01; ***P < 0.001; ****P < 0.0001.

BF.7/BA.5.2 convalescents represented the primary infected population during China’s early Omicron epidemic phase. To evaluate neutralization breadth and durability following BF.7/BA.5.2 infection, we collected sera from convalescents at 14–28 days and 6 months post-recovery for pseudovirus neutralization testing against multiple SARS-CoV-2 variants. Neutralizing antibody analysis demonstrated that 14–28 days post-recovery, sera exhibited robust neutralizing activity against the homologous BF.7 variant (GMT = 285) and responded effectively to prototype (GMT = 956). However, sera demonstrated substantial immune evasion by heterologous variants, with escape fold-changes ranging from 4.57-fold (LP.8.1.1) to 13.36-fold (KP.3). Neutralizing activity against all heterologous variants was significantly reduced compared with homologous strains (**Figure 2A**). Critically, 6-month follow-up data revealed more pronounced immune attenuation, with exacerbated immune evasion by heterologous variants. NB.1.8.1, LP.8.1.1, and BA.3.2 exhibited the strongest immune evasion at 6 months, with fold-changes exceeding 20 (**Figure 2B**). This indicated an accelerated decline in cross-variant neutralization capacity over time. Radar plot analysis visually demonstrated shifts in neutralization response patterns (**Figure 2C**). The GMT against heterologous strains was significantly lower in convalescent sera (14–28 days post-recovery), despite a broader neutralization breadth. Conversely, 6-month sera exhibited markedly narrowed neutralization spectra with generally reduced intensity, indicating limited neutralization capacity against Omicron variants.

Concurrently, we analyzed PBMCs from convalescents at 14–28 days and 6 months post-recovery using ELISpot techniques. Results demonstrated that 14–28 days post-recovery, all convalescents generated robust antigen-specific T cell responses (median > 2,000 SFCs/10^6^ PBMCs) upon stimulation with homologous strain (BF.7 or BA.5.2) S1 peptide pools. Importantly, stimulation with S1 peptide pools of other Omicron variants, including BA.2.75, BQ.1, BQ.1.1, and XBB, also triggered high-level cross-reactivity of comparable intensity (**Figure 2D**). Compared with mock controls, spot-forming cell counts stimulated by all viral antigens showed a significantly higher level, indicating establishment of extensive S protein-specific T cell immunity in convalescents, with no significant inter-variant differences. Six-month ELISpot results further confirmed T cell response durability. In contrast to substantial neutralizing antibody attenuation, T cell immune responses demonstrated a persistent trend. ELISpot analysis showed that S1 peptide pool responses remained elevated (2,500–3,500 SFCs/10^6^ PBMCs) with maintained cross-reactivity against different heterologous strains (including BA.2.75, CH.1.1, BQ.1, BQ.1.1, XBB, and XBB.1.5) (**Figure 2E**). Additionally, RBD peptide pool stimulation triggered significant responses (median > 1,000 SFCs/10^6^ PBMCs) (**Figure 2F**), indicating multiple conserved T cell epitopes within the RBD region capable of inducing long-term immune memory.

### 3.3 XBB convalescents maintained short-term neutralizing responses stability with conserved T cell immunity

**Figure 3:**
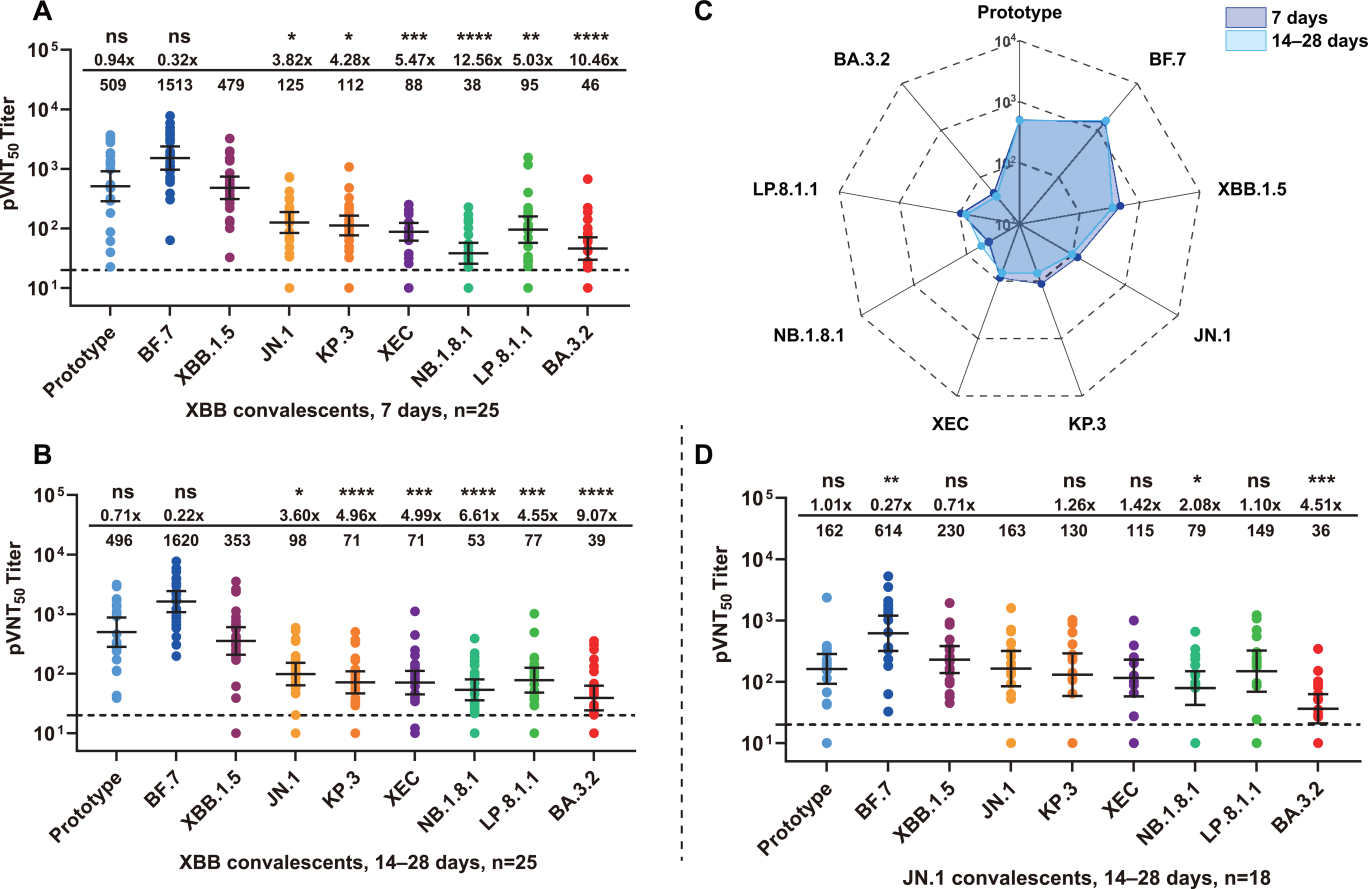
Neutralizing antibody profiles in XBB and JN.1 convalescents. **(A, B)** Serum neutralizing antibody titers in XBB convalescents (n=25). Individual samples are shown as dots, with lines representing GMT and 95% CI. The horizontal dashed line indicates detection threshold (1:20). Values above panels show immune escape fold changes relative to homologous XBB.1.5 and GMT per group. Individual-level temporal trajectories for longitudinal cohorts are shown in **Supplementary Figure 3**. **(C)** Radar plot analysis of neutralizing antibody patterns in XBB convalescents. Neutralization profiles against variants at 7 days (purple shading) and 14–28 days (blue shading) post-recovery demonstrate short-term antibody level stability. Radial axes represent logarithmic GMT scales. XBB convalescents showed relatively stable neutralizing antibody levels shortly after recovery, yet exhibited substantial immune evasion by JN.1 lineage variants and the BA.3.2 variant. **(D)** Serum neutralizing antibody titers in JN.1 convalescents. pVNT_50_ results for sera collected 14–28 days post-recovery (n=18) against multiple variants. Notably, neutralizing activity of JN.1 convalescents against the previously dominant BF.7 variant (GMT = 614) significantly exceeded responses to the JN.1 variant itself (GMT = 162), indicating immune imprinting effects. Additionally, JN.1 convalescents exhibited cross-neutralizing responses to emerging JN.1 lineage variants, while demonstrating substantial escape of antigenically distant variants such as BA.3.2. The “ns” denotes non-significant differences; *P < 0.05; **P < 0.01; ***P < 0.001; ****P < 0.0001.

XBB convalescents represented a second ascent wave of Omicron-infected population in China during mid-2023. We employed identical approaches to assess adaptive immune responses induced by XBB breakthrough infection. Seven days post-recovery, sera demonstrated high neutralizing activity against the homologous strain XBB.1.5 (GMT = 479) while maintaining robust responses against BF.7 (GMT = 1,513) and the prototype (GMT = 509) (**Figure 3A**). Later-emerging variants exhibited obvious immune evasion of neutralizing antibodies, with fold-changes ranging from 3.82–12.56 (*P* < 0.05), among which NB.1.8.1 variant showed the highest escape fold-change (12.56). The 14–28 days follow-up data revealed that although XBB.1.5 titers decreased slightly (GMT = 353). Escape patterns paralleled 7-day data, with sera continuing to exhibit substantial evasion of JN.1 lineage strains, especially NB.1.8.1 (**Figure 3B**). Radar chart analysis intuitively demonstrated near-complete overlap between the two timepoints. Serum neutralizing antibody titers remained relatively stable during short-term recovery but still provided inadequate protection against newly emerging variants (**Figure 3C**).

**Figure 4:**
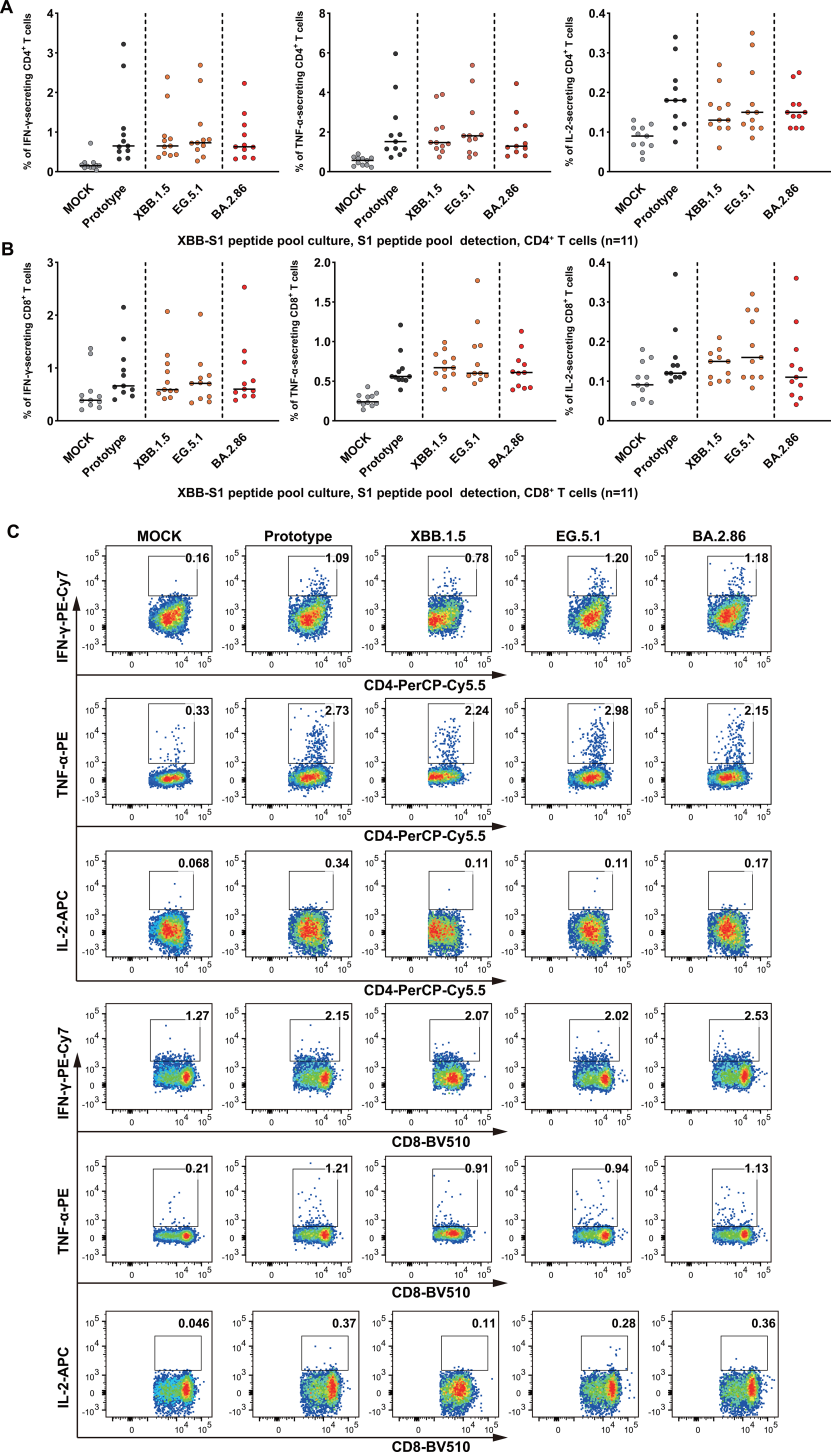
Multifunctional T cell cytokine responses to SARS-CoV-2 variants in XBB convalescents. **(A)** CD4^+^ T cell cytokine secretion analysis. Intracellular cytokine staining assessed IFN-γ, TNF-α, and IL-2 secretion by CD4^+^ T cells from XBB convalescents (14–28 days post-recovery) following stimulation with S1 peptide pools from different SARS-CoV-2 variants. Left, middle, and right panels display percentages of IFN-γ^+^, TNF-α^+^, and IL-2^+^ cells, respectively. Individual samples (n=11) are represented as dots, with horizontal lines indicating medians. **(B)** CD8^+^ T cell cytokine secretion analysis. CD8^+^ T cell cytokine patterns under identical stimulation conditions. Panel layout and statistical analyses mirror **(A)**. **(C)** Representative donor flow cytometry profiles. Multifunctional cytokine secretion patterns from a typical donor following variant S1 peptide pool stimulation. Upper three rows display CD4^+^ T cell IFN-γ^+^, TNF-α^+^, and IL-2^+^ populations, and lower three rows show corresponding CD8^+^ T cell cytokine secretion. Numbers in upper-right corners indicate positive cell percentages. Conditions from left to right: MOCK (unstimulated control), prototype, XBB.1.5, EG.5.1, and BA.2.86 S1 pools stimulations. Results demonstrated XBB convalescent T cells exhibited robust cross-reactivity to diverse SARS-CoV-2 variants. No significant differences were found between the detected variants using the Friedman test and Dunn’s multiple comparison test for pairwise comparisons.

Functional T cell response analysis revealed cross-reactive response characteristics similar to BF.7/BA.5.2 convalescents. ICS analysis following S1 peptide pool stimulation with representative strains (prototype, XBB.1.5, EG.5.1, and BA.2.86) demonstrated that multifunctional cytokine responses (IFN-γ, TNF-α, and IL-2) could be induced in CD4^+^ T cells, with heterologous strain response intensities not significantly different from homologous strain XBB.1.5 (**Figure 4A**). Cross-reactivity was also observed in CD8^+^ T cells. All tested strains effectively activated CD8^+^ T cells and enabled stable cytokine secretion (**Figures 4B, C**). PBMCs stimulated with the S1 peptide pools of the four strains all produced significant secretion of three cytokines, with IFN-γ and TNF-α as the predominant cytokines. These results again confirmed the broad spectrum and cross-reactivity of T cell immunity. T cell functional responses following XBB infection remained relatively stable under different stimulation conditions, indicating that activated T cell epitopes may be relatively conserved across different Omicron lineages.

### 3.4 JN.1 convalescents demonstrated partial cross-protective immunity

We subsequently evaluated humoral immune response characteristics in individuals recently recovered from JN.1 infection. Cross-reactivity analysis against new variants revealed that JN.1 convalescent sera exhibited relatively mild immune evasion patterns against descendant lineage variants. Escape fold-changes ranged from only 1.10–2.08 (**Figure 3D**), significantly lower than levels observed in BF.7/BA.5.2 and XBB groups. Particularly, sera maintained robust neutralizing activity against several JN.1 descendant variants, including KP.3, XEC, and LP.8.1.1 (*P* > 0.05), with the descendant variant NB.1.8.1 exhibiting the highest escape degree (*P* < 0.05). This suggested that infection with recent variants may provide some degree of cross-reactivity against currently circulating variants (**Figure 3D**). Nevertheless, sera demonstrated limited cross-neutralization capability against the heterologous variant BA.3.2, still exhibiting obvious immune evasion (4.51-fold, *P* < 0.001), consistent with the results from other groups and further confirming the special status of BA.3.2 as the most antigenically distant variant in the study. Notably, serum neutralization titers against phylogenetically earlier variants decreased significantly, with GMT of only 162 against the prototype. However, the strongest neutralization response was observed against BF.7 (GMT = 614), potentially reflecting immune imprinting effects or mixed infection backgrounds.

### 3.5 Early Omicron-component vaccines induced cross-reactive responses against the variant panel

**Figure 5:**
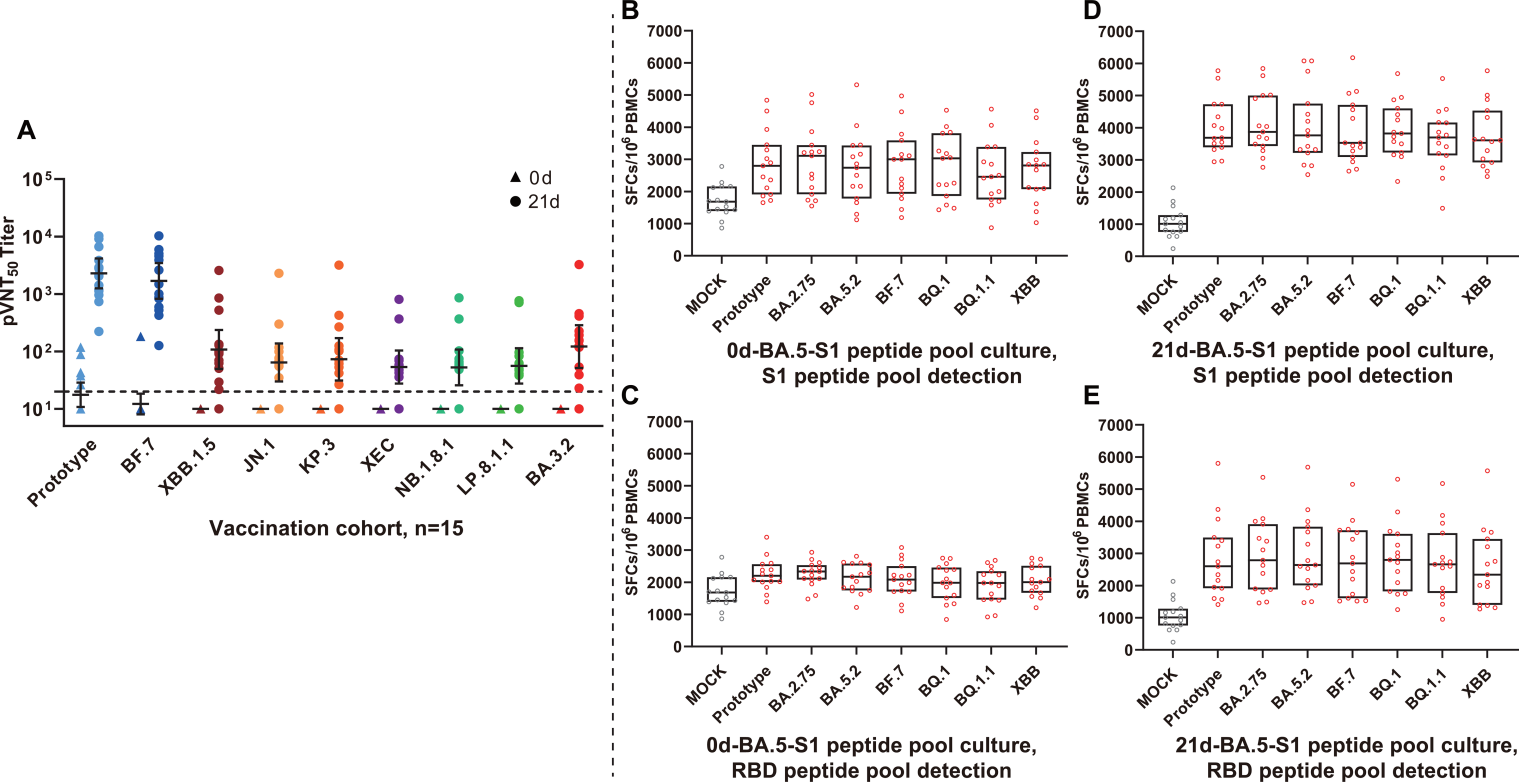
Immune responses elicited by tetravalent recombinant protein vaccination. **(A)** Pre- and post-vaccination neutralizing antibody dynamics. Comparison of pVNT_50_ values in the vaccination cohort (n=15) against different SARS-CoV-2 variants pre-vaccination (0d, triangles) and 21 days post-vaccination (21d, circles). The horizontal dashed line marks detection threshold (1:20). Colors correspond to different variants. Individual samples appear as dots, with lines representing GMT and 95% CI. **(B-E)** T cell response assessment. ELISpot measured T cell reactivity to different variants pre- and post-vaccination. **(B)** and **(D)** present results from cells cultured with BA.5-S1 peptide pools and subsequently detected using S1 peptide pools from other variants at 0d and 21d post-vaccination, respectively. **(C)** and **(E)** show corresponding results cultured with BA.5-S1 peptide pools, then detected using RBD peptide pools from other variants. Data are expressed as SFCs/10^6^ PBMCs. Individual samples are represented as dots, with box plots showing median, interquartile range. MOCK represents unstimulated controls. Vaccination significantly enhanced neutralizing antibody levels 21 days post-vaccination, producing the highest titer against the prototype, BF.7, and a higher titer against XBB.1.5. It also demonstrated detectable cross-neutralizing activity against the subsequently prevalent JN.1 lineage variants and BA.3.2. The vaccine induced broad-spectrum T cell responses with significantly enhanced reactivity to S1 and RBD regions across all variants 21 days post-vaccination compared with baseline.

Beyond natural infection convalescents, we also evaluated immune levels in recipients of a recombinant protein vaccine and systematically analyzed serum neutralizing antibodies and antigen-specific T cell responses in subjects on Day 0 and Day 21 post-vaccination. Pseudovirus neutralization assays revealed that 21 days post-vaccination, vaccine-induced neutralizing antibodies produced detectable responses against all tested strains, with GMTs significantly increased compared with pre-vaccination levels (**Figure 5A**). Serum exhibited the strongest neutralizing activity against prototype (GMT = 2,279) and BF.7 (GMT = 1,688), with GMTs exceeding 1,000, indicating that vaccines can induce robust humoral immunity against homologous or antigenically similar strains. Overall titers against various subsequent and currently prevalent Omicron variants remained retained (GMT range 50–150), and this demonstrated relatively balanced cross-reaction patterns without obvious immune evasion.

ELISpot detection of vaccine-induced T cell responses demonstrated that compared with mock controls, all vaccinated individuals generated robust IFN-γ responses upon stimulation by Omicron variant peptide pools (including BA.2.75, BA.5.2, BF.7, BQ.1, BQ.1.1, and XBB), indicating effective cellular immunity activation by vaccination. For the pre-vaccination (**Figures 5B, C**) and post-vaccination (**Figures 5D, E**), ELISpot comparisons demonstrated that stimulation with both S1 and RBD peptide pools could induce significant T cell response enhancement. Particularly, testing 21 days post-vaccination using S1 peptide pools showed that the median T cell response intensity was comparable to that in natural infection convalescents, reaching 3000–4000 SFCs/10^6^ PBMCs, with no significant differences among variants (**Figure 5D**). This further confirmed high conservation of T cell epitopes within the Omicron lineage. Vaccination successfully activated T cell immunity capable of broadly recognizing various variants.

### 3.6 Antigenic cartography revealed distinct immune evasion patterns and neutralization hierarchy after SARS-CoV-2 variant infections

**Figure 6:**
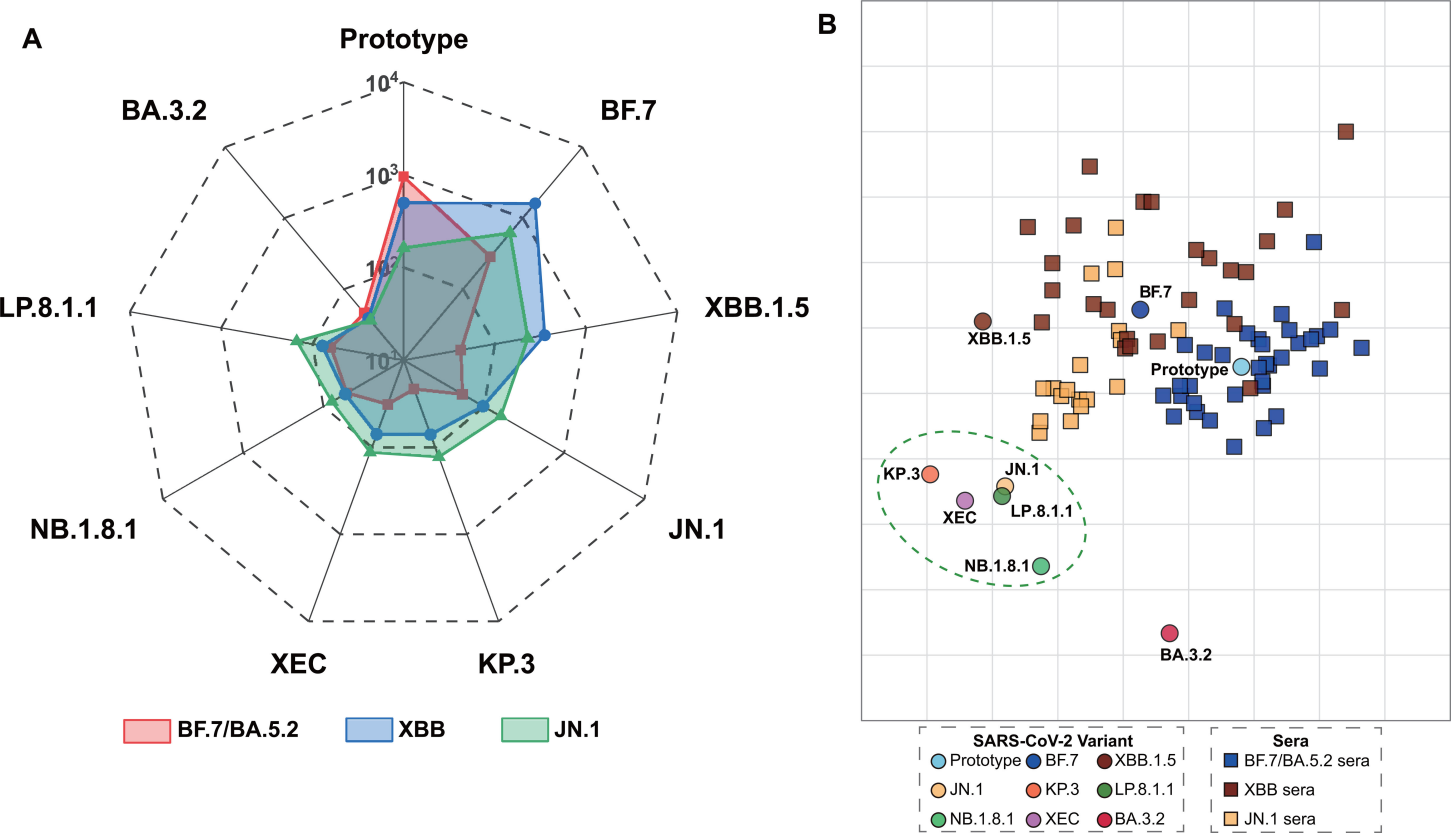
Cross-reactive neutralizing antibody analysis and antigenic cartography across convalescent populations. **(A)** Neutralizing antibody cross-reactivity comparison by radar plot. GMT comparison for sera from BF.7/BA.5.2 (red), XBB (blue), and JN.1 (green) convalescents against nine SARS-CoV-2 variants 14–28 days post-recovery. Radial axes represent logarithmic GMT scales. Results revealed that antibody breadth correlated with infection timing sequence. JN.1 convalescents exhibited the broadest neutralization, followed by XBB convalescents, then BF.7/BA.5.2 convalescents, with BA.3.2 demonstrating the lowest neutralization sensitivity across all convalescent populations. **(B)** Antigenic cartography. Two-dimensional antigenic map constructed from neutralizing antibody data, illustrating antigenic relationships between sera and viral antigens. Squares represent serum samples: blue (BF.7/BA.5.2), brown (XBB), and yellow (JN.1). Circles represent viral antigens, color-coded by variant. The green dashed ellipse encompasses JN.1 lineage variant clustering. One antigenic unit on the map corresponds to a 2-fold change in neutralizing antibody titer. Both X and Y axes represent abstract antigenic dimensions derived from MDS, only the relative Euclidean distances between points reflect actual antigenic relationships. Closer proximity between a serum (square) and virus (circle) indicates higher neutralization efficiency. Greater distance indicates reduced cross-neutralization and antigenic escape. All three convalescent sera clustered near early variant antigens (prototype, BF.7), reflecting immune imprinting. JN.1 sera demonstrated the closest proximity to JN.1 lineage antigens while maintaining cross-reactivity to early antigens. Notably, BA.3.2 exhibited the greatest antigenic distance from all sera, demonstrating maximal immune evasion in this study and warranting continued surveillance.

To systematically evaluate humoral immune response characteristics induced by natural infection with different Omicron variants, we conducted comprehensive antigenic cartography analysis to quantify antigenic relationships between sera and viral variants. Radar chart analysis clearly revealed differences in strain specificity and cross-reactivity. Neutralization levels among the three convalescent groups followed the JN.1 group > XBB group > BF.7/BA.5.2 group (**Figure 6A**). This gradient was highly consistent with the relationship between infection timing and variant evolution. The BA.3.2 variant exhibited the most pronounced immune evasion across all groups, with consistently low neutralizing antibody levels.

Antigenic cartography displayed these relationships in two-dimensional space (**Figure 6B**). Clustering distribution patterns clearly showed evolutionary relationships, with prototype, BF.7, and XBB.1.5 in a central position, while JN.1 and its descendants in a relatively independent antigenic cluster, clearly separated from early BF.7 and XBB lineages. BA.3.2 variant was positioned most distantly from all other variants. Correspondingly, convalescent sera clustered according to their infection backgrounds. BF.7/BA.5.2 sera concentrated near prototype and BF.7, indicating narrow antigenic specificity with limited cross-reactivity against JN.1 lineage variants. XBB sera were intermediately distributed, while JN.1 sera clustered near both JN.1 lineage variants and early antigens, reflecting immune imprinting effects. Notably, BA.3.2 variant was located in marginal areas distant from all serum cluster centers. This indicates substantial antigenic divergence from currently circulating Omicron variants. This consistent escape pattern across all convalescent populations suggests that BA.3.2 possesses unique antigenic characteristics warranting continued surveillance.

## 4 Discussion

**Figure 7:**
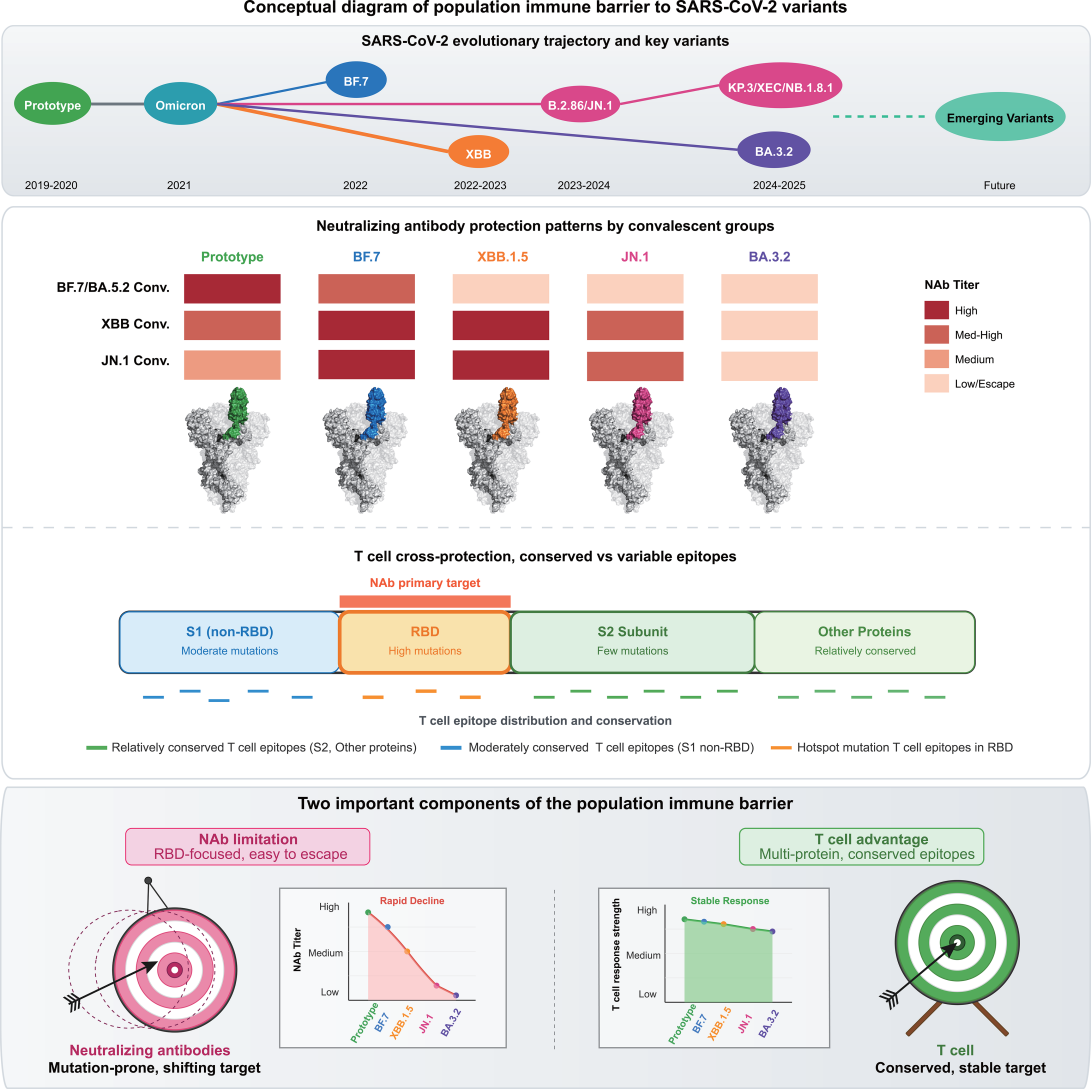
Conceptual diagram of population immune barrier to SARS-CoV-2 variants. Upper panel: SARS-CoV-2 evolutionary trajectory, illustrating temporal progression and phylogenetic relationships from ancestral strains to future variants. Major variants include Omicron (2021), BF.7 (2022), XBB (2022-2023), BA.2.86/JN.1 (2023-2024), and KP.3/XEC/NB.1.8.1 (2024-2025) plus BA.3.2. Dashed arrows indicate potential future emerging variants. Middle panel: Immune protection patterns across convalescent populations. A heat map displays neutralizing antibody titers from BF.7/BA.5.2, XBB, and JN.1 convalescents against representative variants. Color intensity correlates with GMT (dark red = high titer; light colors = low titer/immune escape). Schematic diagram of spike protein structures with highlighted variant-specific RBD regions (color-coded). Viral protein schematics below illustrate T cell cross-protection molecular basis, showing T cell epitope distribution and conservation across viral proteins. S1 non-RBD regions (blue) contain moderate mutations, RBD domains (orange) serve as primary neutralizing antibody targets with frequent hotspot mutations, while S2 subunits (green) and other proteins (light green) remain highly conserved with minimal mutations. Key findings demonstrated that infection timing influenced neutralizing antibody breadth and progressive evasion of subsequent variants, whereas T cell responses maintained stable cross-reactivity. Bottom panel: Two important components of the population immune barrier against emerging SARS-CoV-2 variants. The pink, swaying, suspended target represents the antibody target characterized by being prone to mutation and unstable (left). The green, fixed target represents the T cell target characterized by conserved and stable characteristics (right). Rapid neutralizing antibody decay compromises protection against new variants, while T cell responses sustain long-term cross-protection by targeting conserved epitopes, establishing crucial immune compensation. This study revealed a dynamic interplay between SARS-CoV-2 immune evasion and host protection, emphasizing the importance of T cell-mediated cross-protection in managing viral evolution.

This study characterized immune dynamics across three consecutive Omicron epidemic waves in China, revealing variant-specific neutralizing antibody escape counterbalanced by durable, broad-spectrum T cell cross-reactivity (**Figure 7**). These findings have direct implications for optimizing vaccine strategies and immune surveillance. Our analysis confirmed complex decay kinetics of neutralizing antibodies across different Omicron infection backgrounds. BF.7/BA.5.2 convalescents exhibited rapid antibody decay, with escape fold-changes against heterologous variants progressively increasing over the 6-month observation period. This pattern aligns with previous studies demonstrating significant neutralizing antibody decline within 3–6 months post-infection (47, 48). XBB convalescents maintained relatively stable short-term antibody levels, while JN.1 convalescents demonstrated superior cross-reactivity against descendant lineages. This hierarchical reactive pattern directly reflects the dynamic equilibrium between viral evolution and host immune pressure (49). Some individuals recovering from BF.7/BA.5.2 infections showed increased neutralizing antibody titers against certain variants at 6 months, potentially reflecting subclinical re-exposure or antibody maturation. Individual trajectory analysis (**Supplementary Figure 3**) revealed significant inter-individual heterogeneity, although the group-level trends still clearly showed that homologous immunity remained relatively stable, while cross-reactive immunity gradually weakened. Especially, cross-neutralization against antigenically distant variants was substantially compromised (50).

The enhanced immune evasion capacity of emerging variants stems primarily from cumulative mutations in critical neutralizing antibody epitopes. Notably, BA.2.86 and JN.1 variants, harboring over 30 spike protein mutations, exhibited broad resistance to most monoclonal antibodies, including complete escape from the class III antibody sotrovimab (21, 51). Similarly, the XEC variant (KS.1.1 and KP.3.3 recombined) inherited key mutations T22N and Q493E from parental variants, which are strongly associated with enhanced ACE2 binding affinity and immune evasion (52). Recent studies indicate that emerging variants such as NB.1.8.1 and LP.8.1.1 demonstrate stronger ACE2 receptor binding compared with other JN.1 lineage strains. Particularly, BA.3.2, carrying over 50 mutations relative to BA.3, exhibits unique spike protein conformational changes that reduce ACE2 binding while enhancing immune evasion (53, 54). Antigenic cartography analysis quantitatively confirms these escape patterns, revealing BA.3.2 exhibits the greatest antigenic distance from all convalescent sera tested, highlighting the importance of monitoring variants with substantial antigenic divergence (55, 56). These molecular-level alterations provide mechanistic explanations for the neutralizing antibody escape patterns observed in our study.

A striking observation in JN.1 convalescents was elevated neutralizing activity against BF.7 and XBB.1.5 compared with the homologous strain. This preferential recognition of previously circulating variants suggests complex immune imprinting mechanisms due to broad vaccination (57). Traditional “original antigenic sin” theory posits that initial immune exposure constrains subsequent responses to novel antigens (58, 59), yet this pattern likely reflects preferential recall of vaccination-induced immune memory upon reinfection or subclinical exposure to BF.7 or XBB lineages. It is worth noting that the Chinese population had very little exposure to pre-Omicron variants between 2020 and 2022, and the first large-scale wave of infections only occurred at the end of 2022 with the prevalence of BF.7/BA.5.2. This unique epidemiological trajectory stands in stark contrast to populations in other countries that experienced Alpha and Delta infection waves. The immune imprint against SARS-CoV-2 in the Chinese population is primarily shaped by vaccination, mostly with inactivated vaccines derived from the original strain. The predominantly inactivated vaccine background in our cohort may have shaped these immune dynamics differently compared to other platforms, contributing to the observed imprinting patterns. Although immune imprinting enables rapid recall of previous responses, it may compromise breadth against new variants, partially explaining the consistent immune evasion against BA.3.2 across all groups.

T cell immunity exhibited remarkable stability, maintaining elevated responses 6 months post-recovery with balanced cross-reactivity against all tested variants. This stability is consistent with large-scale analyses demonstrating that 70–90% of CD4^+^ and CD8^+^ T cell epitopes remain conserved across Omicron variants (23, 60). Our focus on the S1 region, which is the most mutation-dense domain of the spike protein, enabled direct assessment of whether accumulating changes in this immunodominant target compromise T cell recognition. The robust cross-reactivity we observed despite substantial S1 sequence divergence indicates that conserved epitopes dominate T cell responses even within this highly variable region. Given the greater conservation in S2/M/N proteins, these regions would be expected to elicit even broader T cell cross-reactivity, as supported by comprehensive epitope mapping studies (61, 62). Crucially, even against variants with the greatest antigenic distances, T cells maintained robust cross-recognition capacity. ICS analysis revealed activation of polyfunctional T cells simultaneously secreting IFN-γ, TNF-α, and IL-2, a characteristic strongly correlated with superior viral control (63). Emerging evidence from animal models supports that T cell immunity can provide substantial protection even when neutralizing antibodies are limited (64, 65), challenging the traditional immunological view that “sterilizing immunity” must rely on neutralizing antibodies.

Beyond inducing robust neutralizing antibody responses, vaccination also elicited high-intensity, broad-coverage T cell immunity. The adjuvant system likely contributed to the robust T cell responses through enhancement of CD4^+^ T cell priming and promotion of Th1-polarized immunity, consistent with the observed multifunctional T cell activation patterns (43). However, the broad T cell cross-reactivity induced by the vaccine is primarily attributed to the multivalent antigen design. The multivalent S protein provides a more comprehensive epitope repertoire, increasing the likelihood of binding to different HLA molecules, thereby enhancing the depth of T cell coverage at the population level. This immune profile of “broad neutralizing antibody coverage with robust T cell backup” aligns with emerging “mosaic vaccine” concepts, suggesting that strategies integrating multiple variant antigenic components may enhance effectiveness (66–68). Previous studies support incorporating diverse antigen components to maximize epitope coverage (69, 70). These findings emphasize the value of developing vaccines targeting conserved T cell epitopes as a more sustainable strategy against rapidly evolving viruses (71).

This study has several limitations. First, sample sizes were determined by convenience sampling during rapid epidemic waves rather than *a priori* power calculations. While this limits definitive conclusions, the approach captured immunologically critical time windows and yielded statistically robust results (*P* < 0.01–0.001). Second, variant identification for some participants relied on epidemiological inference during periods of clear variant dominance (> 90% prevalence) rather than complete sequencing confirmation. This approach is supported by high prevalence rates during recruitment and functional validation through neutralization patterns consistent with the inferred variants. Additionally, the study population was primarily Chinese, with predominantly inactivated vaccine backgrounds, which may limit the generalizability of the findings to populations with different vaccination histories. However, this homogeneity offers insights into immune dynamics relevant to over one billion people globally. Third, immune assessment relied on peripheral blood, while SARS-CoV-2 primarily targets the respiratory mucosa. Mucosal immunity characterization would enhance mechanistic understanding, though standardized virus-specific mucosal assays with adequate sensitivity remain under development (72). Fourth, longitudinal follow-up was not uniformly achieved across all cohorts due to the rapid succession of infection waves and participant availability. The robust 6-month data from the BF.7/BA.5.2 cohort nonetheless demonstrates the feasibility and value of extended monitoring. Finally, T cell assessments did not cover all recent variants due to sample constraints, though evaluation of representative strains enables prediction of responses to related variants through epitope conservation analysis (73).

Immune evasion driven by accumulating mutations in SARS-CoV-2 variants remains a key factor underlying persistent viral transmission. Our findings reveal how the escape of variant-specific neutralizing antibodies is compensated for by sustained cross-reactive T cell immunity. This dual protective mechanism has significant implications for vaccine design and immune surveillance. While neutralizing antibodies provide the first line of defense, their rapid waning and variant-specific nature mean that T cell responses may ultimately prove more critical for long-term population immunity. Vaccine efficacy assessments must integrate T cell immunity with serological indicators, as durable cellular immunity compensates for antibody failure against new variants. Next-generation vaccine development targeting conserved T cell epitopes provides a more sustainable strategy than continuously adapting to evolving antibody targets. Extending booster intervals based on T cell persistence rather than antibody kinetics represents a practical immunization strategy. In summary, our findings demonstrate that cross-reactive T cell immunity establishes a resilient population immune barrier that sustains protection against evolving SARS-CoV-2 variants despite progressive antibody escape.

## Data Availability

The original contributions presented in the study are included in the article/**Supplementary Material**. Further inquiries can be directed to the corresponding authors.
