# Supplementary material for: Broad and robust T cell immunity compensates the population immune barrier against antibody-escaping Omicron variants
Source: Front Immunol. 2026 Jan 30;17:1731340. doi: 10.3389/fimmu.2026.1731340 (PMC12901377; doi:10.3389/fimmu.2026.1731340)
Supplement: Supplementary file 1 [file DataSheet1.pdf]

Supplementary materials

Supplementary Figure 1. Dynamic changes in the composition of SARS-CoV-2 variants in China from December 2022 to August 2024.

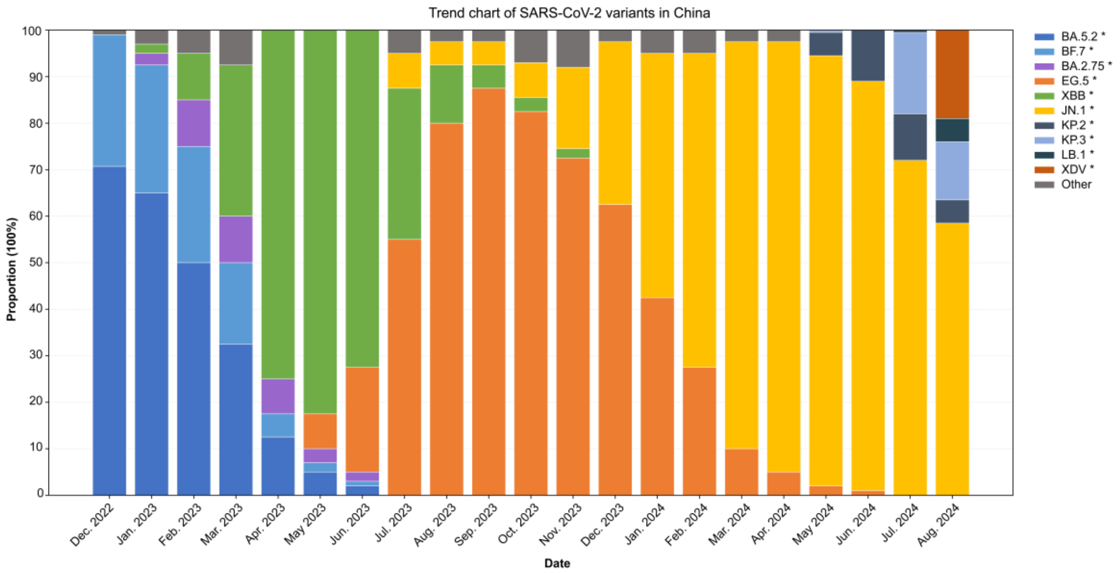

This figure shows the changing trends in the composition of the major SARS-CoV-2 variants circulating in China from December 2022 to August 2024. The vertical axis represents the composition percentage (%) of each variant, and the horizontal axis represents the sampling time (statistics by month). Different colors represent different mutant lineages and their sub-branches, and * indicates that it contains its progeny sub-variants. Data shows that the prevalent SARS-CoV-2 strains in China underwent a clear succession process: BA.5.2 and BF.7 dominated from December 2022 to February 2023; the XBB lineage rapidly replaced the BA.5 series from March to June 2023; EG.5 became the dominant strain from July to December 2023; and the JN.1 lineage continued to grow after its emergence in July 2023, becoming dominant from January to July 2024. This continuous pattern of lineage replacement reflects the virus's ongoing evolution under immune selection pressure and its gradually increasing ability to evade immunity. The subvariant corresponding to the sampling period in this study remained the dominant prevalent variant. The data comes from monitoring data on the official website of the Chinese Center for Disease Control and Prevention.

Supplementary Figure 2. Gating strategy for intracellular cytokine staining (ICS) analysis of T cell responses.

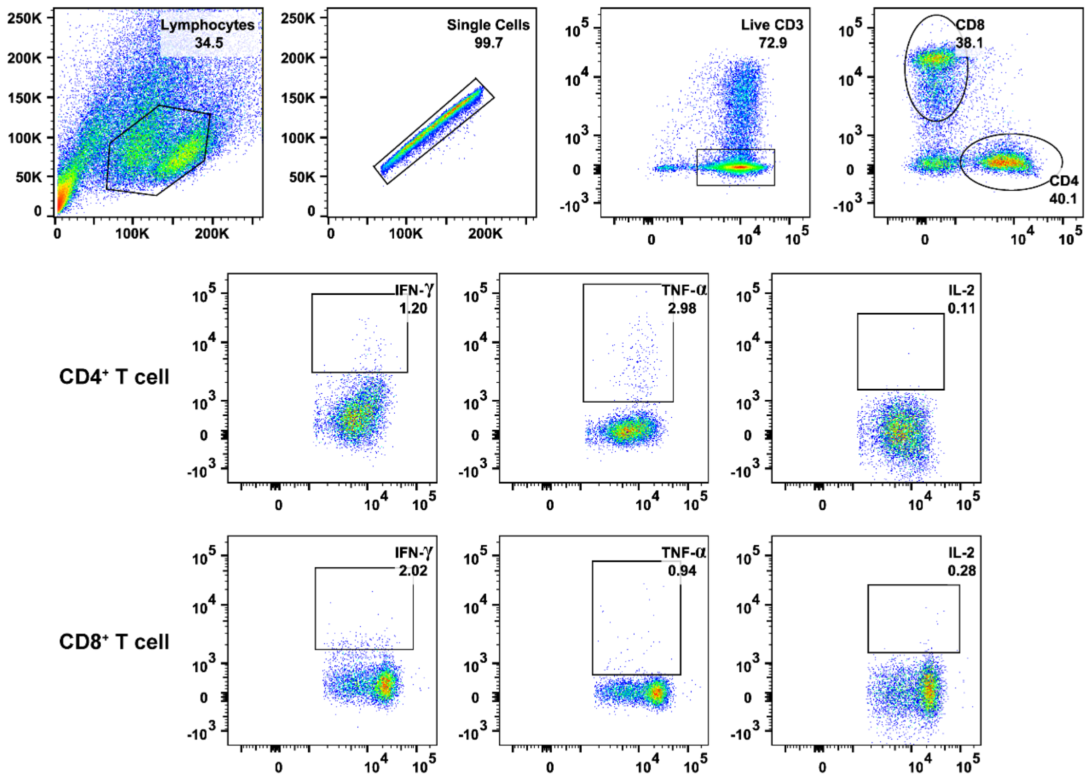

PBMCs were first gated on lymphocytes based on forward scatter (FSC) and side scatter (SSC) characteristics (34.5% of total events), followed by singlet discrimination (99.7% of lymphocytes). Live CD3^{+} T cells were then identified (72.9% of singlets), and subsequently divided into CD4^{+} (40.1%) and CD8^{+} (38.1%) T cell subsets based on surface marker expression. Within each T cell subset, cytokine-producing cells were identified by intracellular staining for IFN-γ, TNF-α, and IL-2. Representative gating shows CD4^{+} T cells producing IFN-γ (1.20%), TNF-α (2.98%), and IL-2 (0.11%), and CD8^{+} T cells producing IFN-γ (2.02%), TNF-α (0.94%), and IL-2 (0.28%) following peptide stimulation. Numbers in each gate represent the percentage of positive cells within the parent population. This gating strategy was consistently applied to all samples to ensure accurate identification and quantification of antigen-specific T cell responses.

Supplementary Figure 3. Individual-level temporal trajectories of neutralizing antibody responses in longitudinal cohorts.

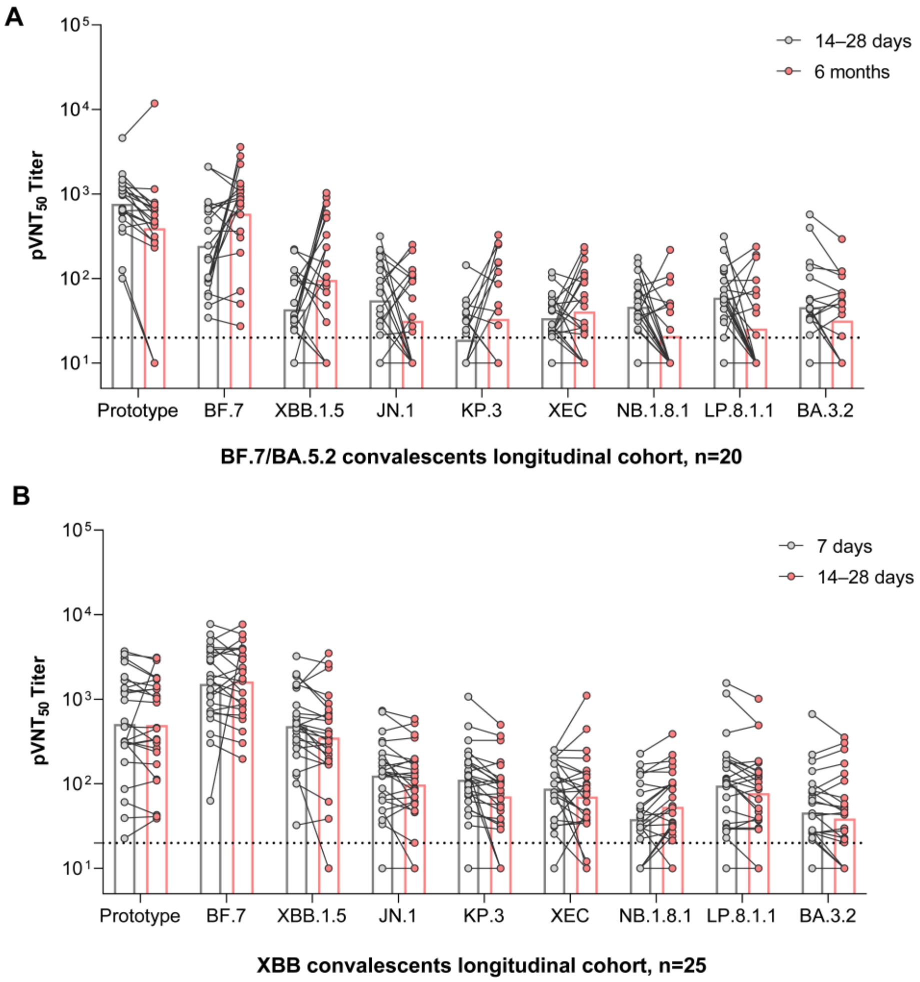

(A) BF.7/BA.5.2 convalescents longitudinal cohort (n=20). Neutralizing antibody titers ($pVNT_{50}$) against indicated SARS-CoV-2 variants at 14–28 days post-recovery (gray circles) and 6 months post-recovery (red circles). Lines connect paired samples from the same participant. (B) XBB convalescents longitudinal cohort (n=25). Neutralizing antibody titers ($pVNT_{50}$) against indicated SARS-CoV-2 variants at 7 days post-recovery (gray circles) and 14–28 days post-recovery (red circles). Lines connect paired samples from the same participant. Individual trajectories demonstrate substantial inter-individual heterogeneity in antibody dynamics. Horizontal bars indicate geometric mean titers (GMT) at each time point. The dotted line indicates the detection limit ($pVNT_{50}$ = 20).

Supplementary Table 1 General demographic characteristics of the participants in the study.

Characteristic		BF.7/BA.5.2 infected (14–28 days) group, n=45	BF.7/BA.5.2 infected (6 months) group [a], n=31	XBB infected cohort [b], n=25	JN.1 infected group, n=18	Vaccination cohort [c], n=15
Sex, n (%)	Male	8 (17.8%)	5 (16.1%)	9 (36%)	9 (50%)	4 (26.7%)
	Female	37 (82.2%)	26 (83.9%)	16 (64%)	9 (50%)	11 (73.3%)
Age	Median (IQR) [d]	37 (28.5, 51.5)	36 (29, 52.5)	28 (25.25, 30)	37.5 (32, 51.25)	25 (24, 28)
	Range	23–61	23–58	23–44	19–71	23–44
Infection history, n (%)	1 time	45 (100%)	27 (87.1%)	7 (28%)	1 (5.6%)	/
	2 times	/	4 (12.9%) [e]	18 (72%)	13 (72.2%)	/
	3 times	/	/	/	4 (22.2%)	/
Vaccination [f], n (%)	0 dose	2 (4.4%)	2 (6.5%)	2 (8%)	2 (11%)	/
	2 doses	4 (8.9%)	4 (12.9%)	2 (8%)	1 (5.6%)	1 (6.7%)
	3 doses	38 (84.5%)	24 (77.4%)	15 (60%)	12 (66.7%)	1 (6.7%)
	4 doses and above	1 (2.2%)	1 (3.2%)	6 (24%)	3 (16.7%)	13 (86.6%)

[a] The BF.7/BA.5.2 cohort initially enrolled 45 participants at the 14–28 day timepoint, of which 31 completed 6-month follow-up (25 from the original cohort, and 6 additionally recruited participants matched for baseline characteristics). Twenty participants were lost to follow-up due to refusal of a second blood draw, loss of contact, or inadequate sample quality. All additionally recruited participants were matched for baseline characteristics (age, sex, disease severity, vaccination history) with the original cohort. The specific sample size for each experimental group is indicated in the figures.

[b] Longitudinal cohort sampled 7 days post-recovery (n=25) and 14–28 days post-recovery (n=25).

[c] Longitudinal cohort sampled 0 days (n=15) and 21 days post-vaccination (n=15). All 15 participants reported no history of SARS-CoV-2 infection at the time of enrollment.

[d] IQR means interquartile range.

[e] During follow-up, all participants were instructed to report any COVID-19-related symptoms. In the BF.7/BA.5.2 longitudinal cohort, 4 participants reported potential symptomatic reinfection during the 6-month follow-up period. Due to resource constraints, systematic virological confirmation was not performed for all participants.

[f] The vaccination status of the subjects is comparable, as most subjects in this study were primarily vaccinated with inactivated vaccines, with the vaccine components being mainly the prototype. Thus, the T cell response characteristics we observed in the study could reflect the natural immune relationship between different variants. This study focused on characterizing variant-specific immune dynamics and temporal evolution rather than comparing responses across diverse vaccination platforms or infection histories.

Inclusion criteria: (1) Age ≥18 years; (2) PCR or antigen-confirmed SARS-CoV-2 infection; (3) infection timing corresponding to periods of dominant variant circulation (confirmed by epidemiological surveillance, Supplementary Figure 1); (4) no respiratory symptoms within 3 months before infection; (5) absence of severe underlying diseases and ability to provide blood samples at designated timepoints.

Exclusion criteria: Immunosuppressive conditions, ongoing immunomodulatory therapy, severe organ dysfunction.

Supplementary Table 2 The NCBI accession numbers of SARS-CoV-2 strains.

SARS-CoV-2 strain	Accession number
Prototype	NC_045512
Alpha	OR611338.1
Beta	OQ341818.1
Gamma	OQ316323.1
Delta	OQ314059.1
BA.1	OQ954674.1
BA.1.1	OQ763014.1
BA.2	OR586925.1
BA.2.12.1	OQ765101.1
BA.2.75	OQ852543.1
CH.1.1	OQ381439.1
BA.5.2	OQ394355.1
BF.7	OQ395842.1
BQ.1	OQ392511.1
BQ.1.1	OQ392501.1
XBB	OQ369094.1
XBB.1.5	OQ602207.1
EG.5.1	OR645809.1
XBB.1.16	OR670646.1
BA.2.86	OR741967.1
JN.1	OR708245.1
XEC	PQ382072.1
XEC.25.1	PV575620.1
NB.1	PQ374341.1
NB.1.8.1	PX089594.1
KP.3	PQ382056.1
KP.3.1.1	PV498133.1
MC.10.1	PQ238450.1
NP.1	PQ862768.1
LP.8	PQ316199.1
LP.8.1.1	PQ791367.1
XFH	PV260870.1
XFG	PV847655.1
LF.7	PQ774529.1
LF.7.7.2	PQ899110.1
LF.7.9	PV397027.1
LF.7.2.1	PQ374363.1
BA.3.2	PV849885.1
